# Combined-modality therapy for pulmonary alveolar proteinosis in a remote setting: a case report

**DOI:** 10.1186/s12890-019-0822-x

**Published:** 2019-03-12

**Authors:** Dacia S. K. Boyce, John W. Lee, Phalgoon Shah, Judy H. Freeman, Matthew C. Aboudara, David C. Hostler

**Affiliations:** 1 Department of Medicine, Tripler Army Medical Center, 1 Jarrett White Rd, Tripler AMC, Hawaii 96859 USA; 2 Department of Medicine, Walter Reed National Military Medical Center, Bethesda, MD USA; 3 Division of Pulmonary, Critical Care & Sleep Medicine, Case Western Reserve MetroHealth Medical Center, Cleveland, USA; 4 Department of Pathology & Laboratory Services, Tripler Army Medical Center, Tripler AMC, USA; 5 Division of Allergy, Pulmonary & Critical Care Medicine, Vanderbilt University Medical Center, Nashville, TN USA

**Keywords:** Pulmonary alveolar proteinosis, Whole lung lavage, Granulocyte macrophage colony-stimulating factor

## Abstract

**Background:**

Pulmonary alveolar proteinosis (PAP) is a rare lung disease characterized by accumulation of phospholipoproteinaceous material in the alveoli. The presentation is nonspecific but typically includes dyspnea; the spectrum of disease includes rapidly progressive hypoxic respiratory failure. Whole lung lavage (WLL) is the treatment of choice in symptomatic PAP, but transient worsening of oxygenation sometimes requires salvage modalities of support such as extracorporeal membrane oxygenation (ECMO). Granulocyte macrophage colony-stimulating factor (GM-CSF) plays a role in the pathophysiology of PAP. We highlight a case of severe PAP treated with exogenous GM-CSF and sequential lobar lavage due to the unavailability of salvage methods of oxygenation.

**Case presentation:**

A 36 year old female was admitted with fevers, chills, and progressive dyspnea. On presentation she was tachypneic, tachycardic, and hypoxemic; labs revealed leukocytosis and lactic acidosis. Chest CT identified diffuse ground glass opacities in a ‘crazy-paving’ pattern. Following intubation due to impending respiratory failure, bronchoscopy with bronchoalveolar lavage was performed. The lavage return stained positive with Periodic Acid Schiff, confirming the diagnosis of PAP. Continued deterioration necessitated treatment; however, at this geographically remote center without ECMO services WLL was judged to carry significant risk. Nebulized GM-CSF was administered without significant improvement. Subcutaneous GM-CSF was administered and isolated subsegmental lavages of the bilateral upper lobes were performed, with rapid improvement in oxygenation. Additional sequential lobar lavage and continued GM-CSF therapy as an outpatient resulted in complete resolution of oxygen requirement and return to normal pulmonary physiology.

**Conclusions:**

The autoimmune form of PAP is the most common, indicating that therapy with GM-CSF may play an important role for many patients. Treatment with WLL may be impractical in some clinical settings due to the expertise and salvage modalities required. Sequential lobar lavage requires less specialized expertise and may incur less risk of refractory hypoxemia. We posit that this combined-modality therapy is ideally suited to geographically-remote centers such as our own.

## Background

Pulmonary alveolar proteinosis (PAP), first described in 1958, is a rare condition affecting the lungs, characterized by defects in surfactant clearance by alveolar macrophages. The inability to catabolize surfactant leads to its buildup in the alveoli, with resultant hypoxemia and increase in the pulmonary alveolar-arterial diffusion gradient [1, 2]. Pulmonary function tests show a restrictive defect, and marked decrease in diffusion capacity for carbon monoxide (DLCO). Lactate dehydrogenase (LDH) may also be elevated, and can be tracked as a marker of disease, as routine lab studies usually do not show any marked abnormalities [1]. The clinical progression of PAP varies widely, with some patients experiencing spontaneous improvement, while others deteriorate to the point of respiratory failure [1–4].

Since its discovery, treatment for PAP has primarily consisted of whole lung lavage (WLL), wherein one lung is intubated while the other is flushed with up to 15 l of saline to clear the lung of surfactant and debris [1, 2, 5]. Risks associated with WLL include hemodynamic instability and refractory hypoxemia requiring the use of extracorporeal membrane oxygenation (ECMO), cardiopulmonary bypass, or hyperbaric oxygen [1, 4–6]. These risks are highest in those critically ill patients already experiencing respiratory failure prior to lavage. Serial lobar lavage is a relatively underreported alternative treatment method which produces clinical and radiological improvement in PAP patients [6–9]. Recent research indicates that granulocyte macrophage colony-stimulating factor (GM-CSF) is also a viable and low-risk treatment option for immune-mediated PAP [10–12].

We present a case of severe PAP treated with both GM-CSF and sequential lobar lavages in order to reduce risk of refractory hypoxemia in a clinical setting where adult ECMO is unavailable.

## Case presentation

A previously healthy 36 year old female of Thai descent presented to the emergency department with a 4 day history of worsening dyspnea and a nonproductive cough. She also reported fevers, chills, and myalgias, for which she had taken both acetaminophen and ibuprofen without relief. The patient had moved to Hawaii in 2013, but had lived until then in her native Thailand. She had not traveled since then, had no sick contacts, no history of tuberculosis or hematologic disease, no unusual occupational exposures, and was not immunocompromised. She was in a monogamous sexual relationship.

**Fig. 1:**
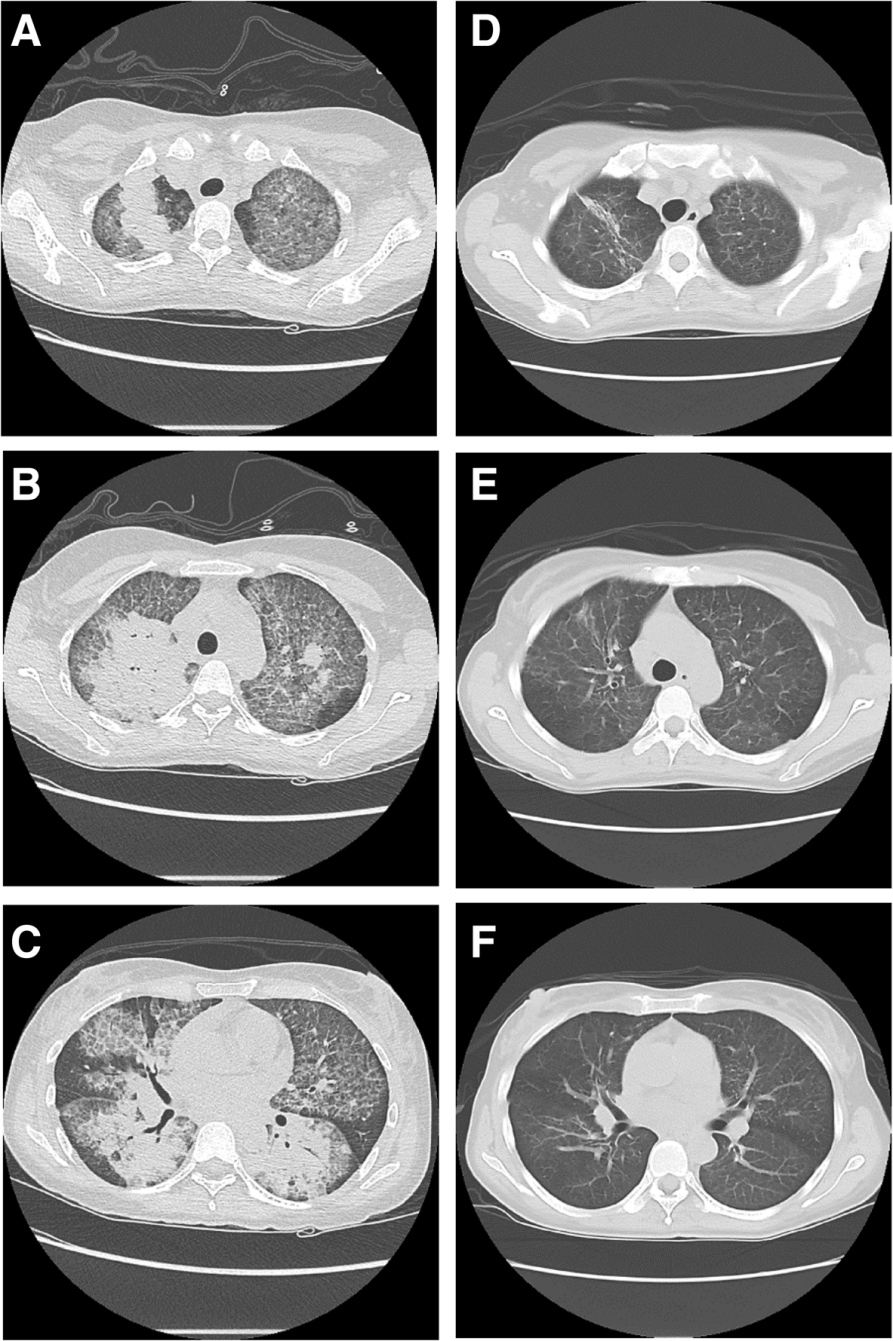
Noncontrast CT chest on presentation and following treatment. **a-c**: representative cross-sectional images of the chest on presentation. **d-f**: matched images following completion of combined-modality therapy with GM-CSF and serial lobar lavage

Upon initial presentation, the patient maintained oxygen saturation over 90% on room air but was tachypneic to > 50 breaths per minute and tachycardic, with signs of accessory muscle use and increased work of breathing. Chest radiograph performed in the emergency department revealed increased interstitial markings and alveolar airspace disease. Non-contrast computed tomography (CT) of the chest demonstrated diffuse smooth interlobular septal thickening, with superimposed areas of ground glass attenuation and peribronchial airspace consolidation (Fig. 1). These nonspecific but significant findings were concerning for acute respiratory distress syndrome (ARDS) and other noncardiogenic causes of pulmonary edema, PAP, atypical infectious processes such as *Pneumocystis jirovecii* pneumonia, alveolar hemorrhage, or drug-induced lung disease.

**Fig. 2:**
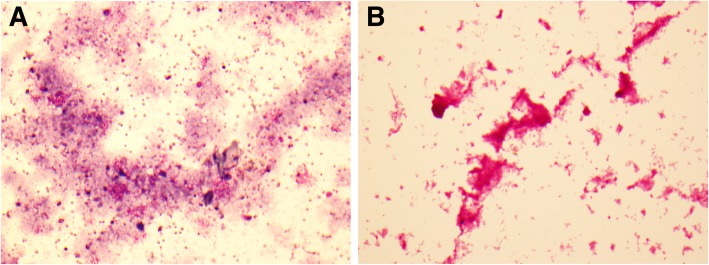
Periodic-Acid Schiff staining of BAL specimen. **a**: Diff Quik stain of BAL fluid demonstrating abundant amorphous granular debris with few macrophages and neutrophils. **b**: Periodic-Acid Schiff (PAS) stain of BAL fluid

Lab abnormalities included microcytic anemia, elevated lactate (2.9 mmol/L, with subsequent 5 h trend to 3.9 mmol/L), mildly elevated procalcitonin (1.91 ng/mL), and lactate dehydrogenase (LDH) of 286 u/L. HIV – 1 + 2 antigen + antibody assay was negative. After volume administration and initial doses of azithromycin and ceftriaxone, the patient was admitted to the intensive care unit. An arterial blood gas drawn shortly after her arrival showed a pH of 7.41, pCO_2_ 24, and pO_2_ 63 on FiO_2_ 0.40. Oxygenation worsened despite empiric antimicrobial therapy, requiring intubation and mechanical ventilation for progressive hypoxic respiratory failure on the first hospital day. Diagnostic bronchoscopy with bronchoalveolar lavage (BAL) was performed; cell count and differential of lavage fluid showed granular amorphous debris with few macrophages and neutrophils. Wright-Giemsa and acid-fast stains of the lavage material were not consistent with *Pneumocystis* or tuberculous pneumonia, respectively; subsequent Periodic Acid-Schiff staining of the amorphous granular material was positive, consistent with PAP (Fig. 2). No findings concerning for hematologic malignancy were seen on peripheral blood smear, lowering suspicion for underlying malignancy. Anti-GM-CSF autoantibody assay was sent and eventually returned positive with a concentration of 7.9mcg/mL, consistent with autoimmune PAP; however, this result was not available for 21 days and thus not useful for clinical decision-making.

WLL was considered, as this is the treatment of choice in symptomatic PAP [1, 2, 7]. However, oxygenation transiently worsens during WLL; when this occurs, salvage methods such as ECMO are typically employed [5]. This patient’s oxygenation was tenuous and ECMO is unavailable in this geographically remote location, so an alternative treatment regimen was devised. The patient was initially treated empirically with aerosolized GM-CSF (dose 250mcg), administered in-line with the endotracheal tube circuit, for two days while awaiting BAL results, but experienced no clinical improvement. Following confirmation of the PAP diagnosis but before anti-GM-CSF antibody results were available, subcutaneous (SQ) GM-CSF (also at 250mcg) was initiated [10]. This yielded a decrease in LDH measurements and improvement in oxygenation such that the patient was successfully extubated on the fourth day of SQ GM-CSF therapy [10–12]. However, she continued to require supplemental oxygen at 4 l per minute at rest and remained profoundly dyspneic. In the days following extubation, her LDH also began to rise, from 381 u/L on her first day of SQ GM-CSF to 536 u/L five days later.

**Fig. 3:**
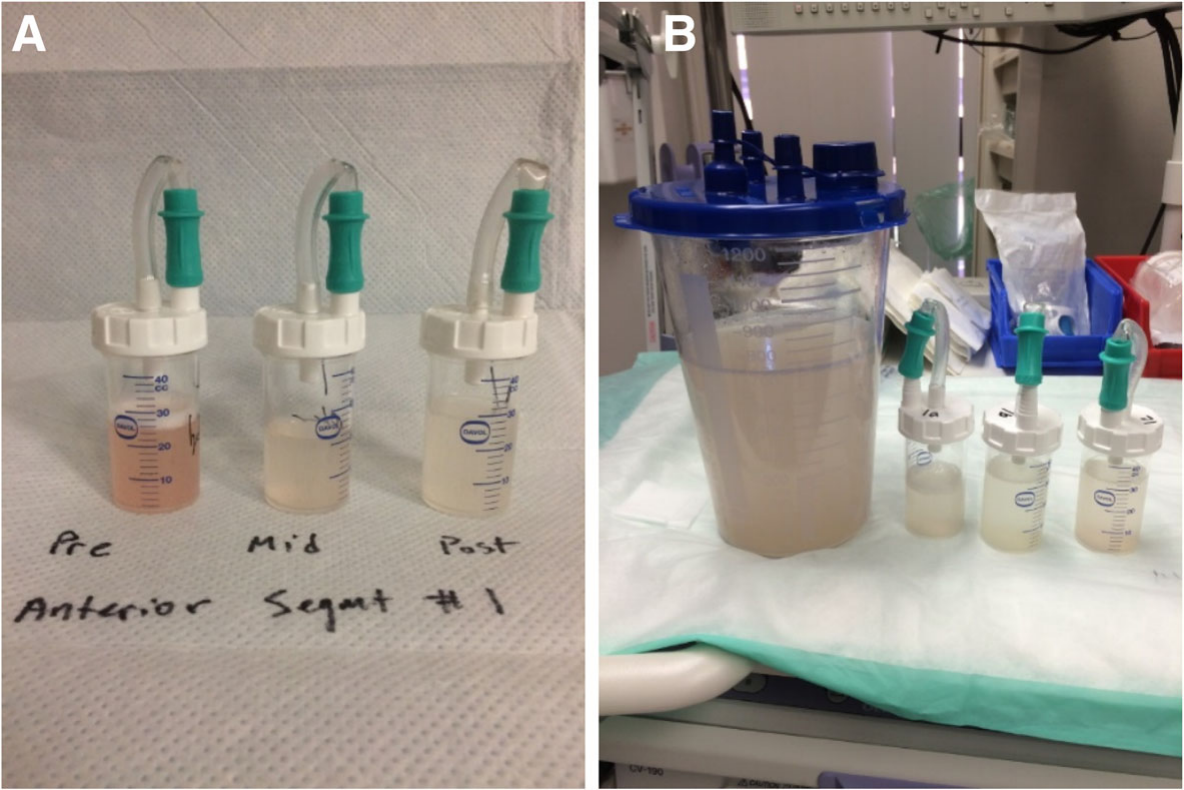
Representative sample of therapeutic lavage fluid. **a**: Initial, mid-procedure, and final lavages from a single segment during the first therapeutic lavage. **b**: Total volume of lavage for a single segment during the second procedure

Following discussions with colleagues in a large academic center, the patient was offered and elected to undergo sequential lobar lavage of the anatomic lobes most severely affected on CT in order to expedite clearance of the alveolar space. During each treatment session, the patient was intubated under general (total intravenous) anesthesia without neuromuscular blockade. An Olympus P-180 videobronchoscope was introduced through the endotracheal tube and wedged into each compatible segmental or subsegmental airway of the lobe being treated. Each anatomic region was lavaged with warmed normal saline solution in increments of 50-60 mL until the return was clear (Fig. 3). The right upper lobe (most radiographically abnormal) was lavaged first and required 1500 mL to return partially-cleared. One week later, the left upper lobe and lingula were lavaged with 4000 mL; an additional week later, segments of the right middle and lower lobes were lavaged with 3500 mL of saline solution. Over the course of three sessions, lavage return was consistently > 85% of instilled fluid.

After each lobar lavage, the patient experienced stepwise improvement in her symptoms, oxygen requirement, and tachypnea. LDH also decreased immediately following her right upper lobe lavage, from 536 u/L to 404 u/L. Repeat imaging showed improvement not only in the areas lavaged, but also in the contralateral lung. The day after her second lobar lavage, she had no oxygen requirement at rest, and denied any shortness of breath even with moderate exertion, although she required 3 l by nasal cannula to maintain saturation > 90% when walking. LDH continued to decrease, to 253 u/L. The patient was discharged shortly thereafter with home oxygen for exertion, and completed a total four week course of SQ GM-CSF. The third lavage was performed on an outpatient basis.

One month after discharge, the patient no longer required supplemental oxygen. Nine months after discharge, surveillance imaging of the lungs showed great improvement in previously visualized ground-glass opacities, with only small areas of residual disease. The patient has not required repeat therapy with either GM-CSF or lavage, and at the time of writing is asymptomatic, with preserved FEV_1_, FVC, and DLCO.

## Conclusions

This patient had clinically and radiographically severe immune-mediated PAP and developed respiratory failure shortly after admission. WLL is undeniably therapeutic in this situation, but also presents high risks for refractory hypoxemia, as the patient must be oxygenated by a single, diseased lung while the other is flooded with saline [1, 2]. The procedure requires deep sedation, generally with neuromuscular blockade, and often requires extreme Trendelenburg – reverse Trendelenburg positioning to recover lavage fluid. These features of WLL place already unstable critically ill patients at risk for hemodynamic instability in addition to failure of oxygenation [5]. Due to the risks involved, the performance of WLL has been largely limited to centers with ECMO capabilities.

Previously published cases have advocated for lobar lavage as a safe alternative to WLL for patients with both severe disease features, who are at risk for hypoxemic or hemodynamic complications, but also for those with mild disease, for whom WLL may not be necessary for relief of symptoms [6–9]. Performing focused lavage allows for lighter sedation, allows oxygenation and ventilation in other segments of the treated lung, and may reduce risk for infection [1, 6]. BAL is not without its limitations; the process is time-consuming and patients often require repeated lavages, as in this case. However, this is not dissimilar from WLL, as PAP patients often require two rounds of WLL before entering remission [1, 3, 10]. Simultaneous treatment with subcutaneous GM-CSF contributed to a rapid recovery in this patient, as evidenced by her radiographic improvement in disease areas which had not undergone BAL.

Although GM-CSF has not been shown to be universally successful in treatment of PAP, its benign side effect profile makes it a useful first line treatment in patients with idiopathic PAP, especially in severe cases where a combined treatment modality can provide an equally efficacious outcome without the risks inherent in WLL [10–12]. GM-CSF produced a 43% response rate in a 2001 case series of 14 patients, but to date no studies have evaluated its efficacy on a larger scale [10–12]. Despite its lower response rate, patients with a positive response who continued long-term GM-CSF treatment experienced no serious toxicity or adverse effects.

In nearly all cases of idiopathic PAP where it is utilized, GM-CSF has been an alternative, rather than an adjunct, therapy to WLL [11]. To our knowledge, this is the first case to utilize stepwise GM-CSF treatment with subsequent serial lobar lavage for treatment of PAP. This treatment regimen has the advantage over WLL in that it can be performed without increased risk for refractory hypoxemia and hemodynamic instability. This obviates the need for ECMO, which in many centers is not available, while maintaining equal effectiveness and long-term outcomes to WLL [6, 7]. WLL, despite its status as treatment of choice in idiopathic PAP, does not treat the underlying cause of immune-mediated disease [3]. As patients with PAP are at increased risk for severe or opportunistic pulmonary infections, lobar lavage in combination with GM-CSF treats both the most dangerous symptom and the underlying cause of the disease in the patients with the highest risk for complications.

## Abbreviations

ARDS: Acute respiratory distress syndrome
BAL: Bronchoalveolar lavage
CT: Computed tomography
ECMO: Extracorporeal membrane oxygenation
GM-CSF: Granulocyte macrophage colony-stimulating factor
LDH: Lactate dehydrogenase
PAP: Pulmonary alveolar proteinosis
SQ: Subcutaneous
WLL: Whole lung lavage
